# Untargeted metabolomics unravel serum metabolic alterations in smokers with hypertension

**DOI:** 10.3389/fphys.2023.1127294

**Published:** 2023-03-02

**Authors:** Yang Shen, Pan Wang, Xinchun Yang, Mulei Chen, Ying Dong, Jing Li

**Affiliations:** ^1^ Department of Nephrology, Beijing Chaoyang Hospital, Capital Medical University, Beijing, China; ^2^ Heart Center and Beijing Key Laboratory of Hypertension, Beijing Chaoyang Hospital, Capital Medical University, Beijing, China

**Keywords:** smoking, hypertension, metabolomics, LC/MS, gut microbiota

## Abstract

**Background:** Cigarette smoking is an important environmental risk factor for cardiovascular events of hypertension (HTN). Existing studies have provided evidence supporting altered gut microbiota by cigarette smoking, especially in hypertensive patients. Metabolic biomarkers play a central role in the functional potentials of the gut microbiome but are poorly characterized in hypertensive smokers. To explore whether serum metabolomics signatures and compositions of HTN patients were varied in smokers, and investigate their connecting relationship to gut microbiota, the serum metabolites were examined in untreated hypertensive patients using untargeted liquid chromatography-mass spectrometry (LC/MS) analysis.

**Results:** A dramatic difference and clear separation in community features of circulating metabolomics members were seen in smoking HTN patients compared with the non-smoking controls, according to partial least squares discrimination analysis (PLS-DA) and orthogonal partial least squares discrimination analysis (OPLS-DA). Serum metabolic profiles and compositions of smoking patients with HTN were significantly distinct from the controls, and were characterized by enrichment of 12-HETE, 7-Ketodeoxycholic acid, Serotonin, N-Stearoyl tyrosine and Deoxycholic acid glycine conjugate, and the depletion of Tetradecanedioic acid, Hippuric acid, Glyceric acid, 20-Hydroxyeicosatetraenoic acid, Phenylpyruvic acid and Capric acid. Additionally, the metabolome displayed prominent functional signatures, with a majority proportion of the metabolites identified to be discriminating between groups distributed in Starch and sucrose metabolism, Caffeine metabolism, Pyruvate metabolism, Glycine, serine and threonine metabolism, and Phenylalanine metabolic pathways. Furthermore, the observation of alterations in metabolites associated with intestinal microbial taxonomy indicated that these metabolic members might mediate the effects of gut microbiome on the smoking host. Indeed, the metabolites specific to smoking HTNs were strongly organized into co-abundance networks, interacting with an array of clinical parameters, including uric acid (UA), low-denstiy lipoprotein cholesterol (LDLC) and smoking index.

**Conclusions:** In conclusion, we demonstrated disparate circulating blood metabolome composition and functional potentials in hypertensive smokers, showing a linkage between specific metabolites in blood and the gut microbiome.

## 1 Introduction

Overwhelming evidences regarding the consequences of smoking have shown that cigarette smoking powerfully enhanced the risks of all-cause mortality, cardiovascular mortality and major adverse cardiovascular events (Chi et al., 2022; Thiravetyan and Vathesatogkit, 2022). Tobacco smoking and even second-hand exposure has been demonstrated to be associated with cardiovascular risk as well as the development of hypertension (HTN) (Dikalov et al., 2019; Bernabe-Ortiz and Carrillo-Larco, 2021). In fact, there has been a growing interest on investigating the role of tobacco consumption on HTN. Hypertensive smokers have been suggested to be more likely to develop malignant or renovascular HTN than non-smokers (Virdis et al., 2010) Cigarette smoking has been shown to raise the daytime and average 24-h blood pressure (BP) and heart rate in treated hypertensive patients (Ohta et al., 2016). Moreover, smokers with HTN were observed to exhibit higher proportion of left ventricular hypertrophy and worse BP control than non-smokers (Journath et al., 2005).

Gut microbiome has emerged as a research hotspot in cardiovascular diseases during the recent years. Bacterial genera and species were reported to be altered in smokers and HTN patients, respectively (Aguilar, 2017; Lee et al., 2018; Nakai et al., 2021). Aberrant microbial community and imbalanced composition and function of gut microbes were indicated to be a consequence of cigarette smoking (Shanahan et al., 2018; Bai et al., 2022), but also as a crucial contributor to disrupting metabolic processes and leading to hypertensive disorders (Li et al., 2017; Yan et al., 2020; Avery et al., 2021). Notably, the metabolites transporting into bloodstream acted as an important bridge for the linkage between gut microbiota and host pathology and physiology (Guest et al., 2016). Past studies have attempted potential alterations in gut microbial functionality in HTN subjects related to smoking status. One important aspect was to examine the varied metabolic functions of gut microbiome in hypertensive cigarette smokers as compared with non-smokers (Wang et al., 2021). It was found that the gut microbiota was disordered among smoking HTN patients, with lower microbial α-diversity and significant difference of β-diversity on axes. In addition, dramatic shifts in the intestinal composition at genus and species levels were found among smokers with HTN, including reduced enrichment of *Phycisphaera* and *Clostridium asparagiforme* (Wang et al., 2021). For another, studies have shown that the smoke-induced gut microbiota dysbiosis impaired gut metabolites directly (Bai et al., 2022). They showed increased bile acid metabolites, especially taurodeoxycholic acid in the colon of mice after smoke exposure (Bai et al., 2022). In addition, the serum metabolome of smoking patients has been identified to differ from that of non-smoking individuals (Xu et al., 2013; Zhang et al., 2022). For instance, Xu et al. indicated that compared with former and non-smokers, in male current smokers, the concentrations of four unsaturated diacyl-phosphatidylcholines (PCs) and five amino acids (arginine, aspartate, glutamate, ornithine and serine) were increased, while three saturated diacyl-PCs, one lysoPC and four acyl-alkyl-PCs, as well as kynurenine showed lower content. Furthermore, higher levels of carnitine and PC aa C32:1, and a lower level of hydroxysphingomyeline [SM (OH)] C22:2 were found in female current smokers (Xu et al., 2013). However, due to the limited number of studies, more investigations are essential to uncover the interrelationship between altered metabolic features and gut microbiota within smokers with HTN.

In an attempt to fully explore the metabolite profiles, and investigate the specific interactive links between metabolites in circulation and the gut microbiome in hypertensive smokers, we employed untargeted liquid chromatography-mass spectrometry (LC/MS) analysis on un-medicated smoking or non-smoking individuals within the clinical context of HTN.

## 2 Materials and methods

### 2.1 Study participants recruitment

The study was conducted according to the guidelines of the Declaration of Helsinki, and approved by the Ethics Committee of Beijing Chaoyang Hospital. All applicable institutional regulations regarding the ethical use of information and samples from human participants were abided, and signed informed consent for the survey have been received from each individual prior to data collection.

The participants were enrolled from our previous study cohort in China (Li et al., 2017) All the individuals wre ethnic Han from employees of the Kailuan Group Corportion of Tangshan city, with simial and stable life-time environment of residence as well as dietary habit. Individuals were excluded if they had suffered from serious cancer, heart or renal failure, stroke, peripheral artery disease or immunodeficiency disorders. All the patients were newly diagnosed hypertensive patients prior to antihypertensive treatment, and none of them have been exposed to antibiotics, probiotics, statin, aspirin, insulin, metformin or nifedipine, etc. Including medicinal herbs during the last 2 months prior to sample collection.

HTN patients with complete information for smoking, including duration and amount for tobacco consumption, as well as smoking coefficient were included in the current research. HTN was diagnosed as systolic BP ≥ 130 mm Hg and/or diastolic BP ≥ 80 mm Hg according to the 2017 ACC/AHA guidelines (Whelton et al., 2017). The measurement of BP was executed by professional nurses or physicians with a random-zero mercury column sphygmomanometer, and subjects were in a sitting position. At every 5 minutes intervals, the BP readings were recorded repeatedly three times, the average of which was regarded as the formal data. Patients having consumed >1 cigarette/day for more than half a year were considered to be smokers as we described previously (Wang et al., 2021), and non-smokers were individuals without a history of tobacco use.

There were 32 non-smokers with HTN (HTN-NS), and 30 cigarette smokers with HTN (HTN-S) in this study. Relevant demographic and clinical profiles such as age, sex, height, weight, body mass index (BMI), fasting blood glucose, total cholesterol, triglyceride, etc. of participants were collected.

### 2.2 Serum sample collection and preparations

Peripheral vein blood was collected from all recruited participants under a fasting state with vacuous tubes, and then separated into serum through centrifugation at 3,000 rpm, 4°C for 10 min. Each aliquot of the obtained serum samples was placed at −80°C and stored until further procedure. For sample preparations before metabolomics determination, serum samples were thawed under room temperature, mixed with 80% methanol and 2.9 mg/mL DL-o-Chlorophenylalanine, vortexed for 30 s and centrifuged at 12,000 rpm and 4°C for 15 min. The supernatant of the solution was proceeded for ultrahigh-performance liquid chromatography with LC/MS detection.

### 2.3 Untargeted metabolome profiling

LC/MS determination was conducted on the platform (Thermo, Ultimate 3000LC, Orbitrap Elite) with Hypergod C_18_ Column. The conditions for chromatographic separation was at 40°C, and 0.3 mL/min for the flow rate, with water+0.1% formic acid, and acetonitrile+0.1% formic acid, respectively. The temperature for automatic injector was at 4°C. For ES + mode, the total ion chromatograms of samples were obtained under 300°C for heater temperature, 45 arb for sheath gas flow rate, 15 arb for aux gas flow rate, one arb for sweep gas flow rate, 3.8 kV spray voltage, 350°C for capillary temperature, and 30% S-Lens RF level. While ES- mode was performed with spray voltage at 3.2 kV and S-Lens RF level at 60%. Peaks were extracted from the raw data and analysis was preprocessed with SIEVE software (Thermo). The data of total ion current was normalized and information for features, including retention time, compound molecular weight, and peak intensity were obtained (Dunn et al., 2011).

### 2.4 Multivariate analysis

Multivariate statistical analyses were conducted based on the serum metabolome composition with SIMCA software (V14.1, Sartorius Stedim Data Analytics AB, Umea, Sweden) to discriminate HTN-S patients from HTN-NS individuals. Firstly, principal component analysis (PCA) as an un-supervised analysis, was carried out to produce new characteristic variables from metabolite variables through linear combination according to weights, and further classify distinct group of samples with the obtained variables (Wiklund et al., 2008). Besides, partial least squares discrimination analysis (PLS-DA) as a supervised analysis has been the most frequently used method for classification in metabonomics data analysis. Regression model was combined with dimension reduction in PLS-DA and discriminant analysis was performed based on regression results with discriminant thresholds (Aggio et al., 2010). Orthogonal partial least squares discriminant analysis (OPLS-DA) was performed to exclude the metabolic orthogonal variables which are not related to classification variables, and analyze the non-orthogonal variables and orthogonal variables respectively (Trygg and Wold, 2002). For annotation strategies, the m/z values and mass of compounds were matched to the featured peaks in the METLIN database and the metabolites were identified. METLIN database enhances accurate quantification and facilitates it to more effectively use the data in metabolite databases (Tautenhahn et al., 2012; Zhu et al., 2013; Alseekh et al., 2021).

### 2.5 Metabolite pathway identification

The differentially expressed compounds between groups were annotated to be involved in metabolic pathways according to Kyoto Encyclopedia of Genes and Genomes (KEGG) Pathway database (http://www.kegg.jp/kegg/pathway.html) (Kanehisa et al., 2016). By both enrichment analysis and topological analysis of the pathways matched with metabolites, key KEGG pathways mostly correlated with the metabolites were detected.

### 2.6 Gut microbial genera and species identification

The metagenomic sequencing, gene catalog construction, taxonomic annotation and abundance profiling of gut microbes at genus and species levels were performed as described in our previous studies (Li et al., 2017; Wang et al., 2021). The whole metagenome shotgun sequencing data of the specimens evaluated in present study have been previously deposited in a public dataset at the EMBL European Nucleotide Archive underthe BioProject accession code PRJEB13870.

### 2.7 Statistical analysis

Subject characteristics were quantitatively described with mean and SD. For continuous variables, range was shown and count and percent prevalence were summarized for categorical variables. The relative abundances of metabolic members from smoking individuals with HTN were compared to non-smoking controls. Z-score was calculated based on the mean and standard deviation of the data set. Z-score=(x−μ)/σ, where x was a specific score, *µ* was the mean, and *s* was the standard deviation (Sreekumar et al., 2009). For univariate analysis, *p* < 0.05 was defined to reach statistical significance in two-tailed Student’s t-test. Metabolic compounds with *p* < 0.05, and variable importance in the projection (VIP) > 1 for the first principal component of OPLS-DA model, were regarded as statistically different between groups. The VIP and the t-tests are two popular strategies for metabolic biomarker selection. The VIP criterion is to infer biomarkers from the multivariate models and the *t*-test aims at selecting them in a univariate mode. We used the multivariate VIP (VIP >1) in conjunction with univariate *t*-test (*p* < 0.05) to identify discriminatory metabolite in the current work, as other investigators performed previously (Chen et al., 2022; Han et al., 2022). A significance threshold of one for the VIP was suggested to lead to much improved enrichment of true positives in the selection (Franceschi et al., 2012; Saccenti et al., 2014). A cutoff at *p* < 0.05 in univariate *t*-test has been frequently adopted by researchers (Chen et al., 2022; Han et al., 2022; Wang et al., 2022). Spearman’s correlation analysis was performed to evaluate the interactions among clinical measures, smoking-related serum metabolites, and HTN-S-related gut microbial composition. The cutoff for correlation coefficient was ≥0.3 or ≤ −0.3, and *p* values were <0.05. The visualization of multiomics correlations was performed using the OmicStudio tools (https://www.omicstudio.cn/tool) with igraph package (version 1.2.6) in R (version 3.6.3). Partial least squares structural equation modeling (PLS-SEM) (Nitzl et al., 2016) was performed with the Smart-PLS three software. The ratio of indirect-to-total effect, variance accounted for (VAF) score, which determines the proportion of the variance explained by the mediation process, was used to examine the significance of mediation effect. Random forest analysis was performed using the random forest package in R to predict the individuals as HTN-NS or HTN-S based on their profiles of genera, species and metabolites. Variable importance by mean decrease in Gini index was calculated for the random forest models. Furthermore, the receiver operation characteristic (ROC) curves for genera, species and metabolites were applied to distinguish the individuals with HTN-S from HTN-NS, statistical significance determined by applying the method of DeLong et al. using MedCalc version 11.4.2.0 (DeLong et al., 1988).

## 3 Results

### 3.1 Biochemical characterization of patients

The measurements of the anthropometric and biochemical data of the study participants were presented in Table 1. The average age of hypertensive smokers was 50.9, and non-smokers mainly aged at 53.7 years. As expected, the duration, amount and coefficient of smoking were significantly higher in the HTN-S group as compared with the non-smoking HTN group. Other baseline clinical characteristics between HTN-NS and HTN-S in systolic blood pressure (SBP), diastolic blood pressure (DBP), Fasting blood glucose and blood lipid index, etc. were similar.

**TABLE 1:** General characteristics of study participants.

Characteristics	HTN-NS	HTN-S	*p*-value
Number	32	30	—
Age, years	53.7 ± 6.2	50.9 ± 4.8	0.053
Male/female sex	29/3	30/0	0.239
Smoking duration (year)	0	25.0 (10.0–32.0)	<0.001
Smoking amount (cigarette/day)	0	10.0 (7.0–20.0)	<0.001
Smoking coefficient (year cigarette/day)	0	270.0 (100.0–500.0)	<0.001
Systolic BP, mmHg	140.0 ± 15.7	138.3 ± 18.3	0.698
Diastolic BP, mmHg	89.3 ± 9.4	89.6 ± 9.4	0.908
HR, bmp	72.4 ± 6.1	71.6 ± 9.5	0.691
Body mass index, kg/m^2^	25.4 ± 3.4	25.6 ± 2.6	0.756
Uric acid, μmol/L	368.00 (275.50–420.00)	360.50 (303.00–400.00)	0.688
Creatinine, μmol/L	71.50 (61.00–91.10)	70.00 (63.00–76.00)	0.356
Fasting blood glucose, mmol/L	5.57 ± 0.60	5.64 ± 0.96	0.765
Total cholesterol, mmol/L	5.39 ± 0.96	5.35 ± 0.74	0.880
Triglyceride, mmol/L	1.32 (0.95–2.06)	1.76 (1.17–2.35)	0.120
HDLC, mmol/L	1.35 ± 0.27	1.27 ± 0.27	0.248
LDLC, mmol/L	2.58 ± 0.79	2.47 ± 0.58	0.562
TBIL, μmol/L	13.95 (10.95–19.05)	13.95 (11.00–18.50)	0.933
Hemoglobin, g/L	157.50 (150.00–161.00)	161.00 (156.00–164.00)	0.058
Blood platelet, *10^9/L	222.00 (190.00–251.50)	220.00 (185.00–255.00)	0.882
White blood cell, *10^9/L	6.20 (5.35–6.85)	6.30 (6.00–7.20)	0.244

NS: non-smokers; S: smokers; HTN: hypertension; BP: blood pressure; HR: heart rate; LDLC: low-density lipoprotein cholesterol; HDLC: high-density lipoprotein cholesterol; TBIL: total bilirubin.

### 3.2 Characterization of the serum metabolomic profiling

**FIGURE 1:**
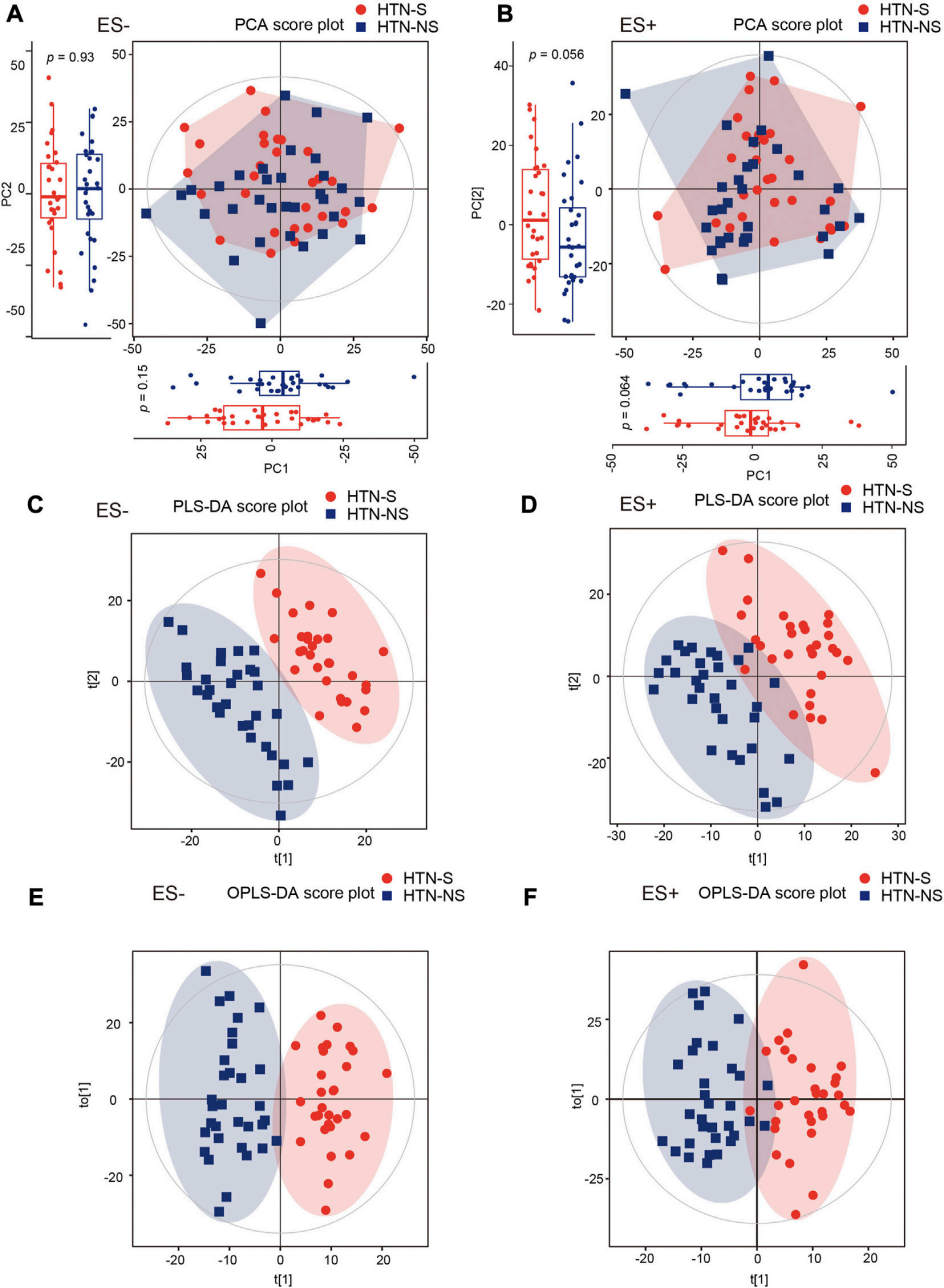
Smoking or not conduced to dissimilarity of serum metabolic community in hypertensive patients. **(A, B)**, Changes of overall metabolic signatures in hypertensive smokers as compared with non-smokers were identified with PCA score plots based on negative (ES−) and positive (ES+) mode, respectively. Hotelling’s T-squared ellipse indicated 95% confidence interval. The distributions of samples in PC1 and PC2 coordinate axis were further shown with box plots. The first and third quartile (25th and 75th percentile) was expressed with boxes, and median was represented with the inside line. Whiskers extend 1.5 times the inter quartile range from the outer bounds. p values were derived from two-tailed Student’s t-test. **(C, D)**, PLS-DA score plots of both ES- and ES + mode serum metabolomic data from HTN-S patients and HTN-NS controls. Subjects from each group were completely separated. **(E, F)**, Score plot of the OPLS-DA showing disparate metabolic profiling in HTN patients smoking cigarette or not. OPLS-DA method is a supervised multiple regression analysis for identifying discernible patterns between different groups. HTN-S, smokers with HTN; HTN-NS, non-smokers with HTN.

Serum metabolite assessment was performed with LC/MS, and 3,436 and 4,079 metabolic peaks were detected within ES+ and ES- mode, respectively. Across over 7,000 distinct metabolic features obtained, we identified 561 endogenous small-molecule compounds. The overall discrepancy in the serum metabolome profiles between HTN-S and non-smokers were assessed through multivariate statistical analysis including PCA, PLS-DA and OPLS-DA in Figure 1. In both positive and negative modes, the PCA score plots showed that samples in HTN-S were mixed with those in HTN-NS group (Figures 1A,B). It was noted that, marginal different distributions of samples from HTN-S and HTN-NS were detected in PC1 and PC2 axis under ES + mode. Discriminant analysis with PLS-DA under positive and negative ionic mode revealed significantly separated clustering of hypertensive smokers and non-smoking controls, respectively (Figures 1C,D). And plots obtained in the OPLS-DA models further validated the two distinct clusters of subjects from disparate groups in ES− and ES+ (Figures 1E,F).

### 3.3 Metabolite markers for discriminating HTN-S from HTN-NS

**FIGURE 2:**
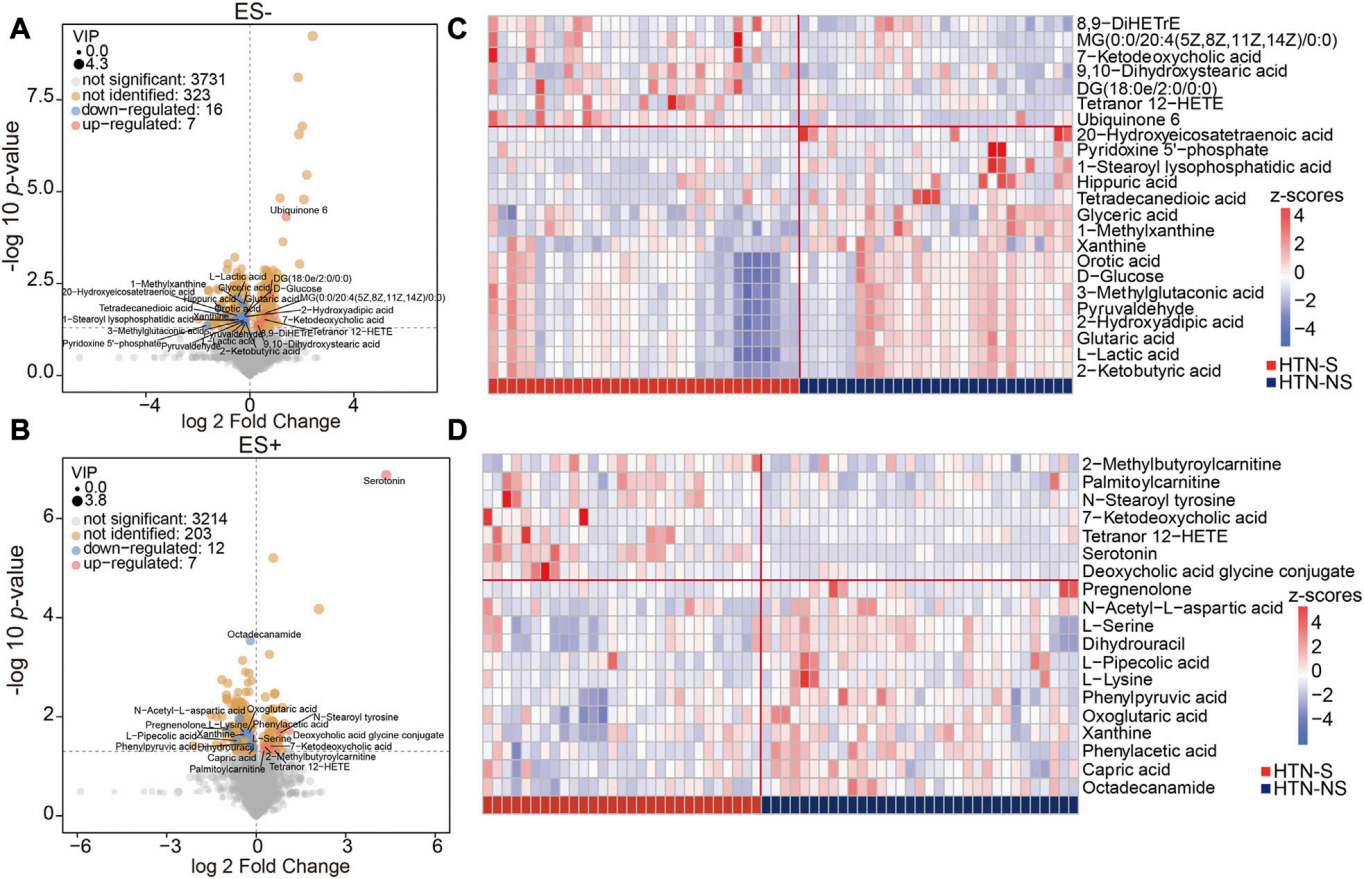
Identification of the differential metabolites associated with smoking and non-smoking HTN. **(A, B)**, Volcano plots showing the important metabolites concluded from the OPLS-DA model using a threshold of variable importance for the projection (VIP) > 1. A is based on metabolomics data in ES- mode, and B is in ES- mode. Comparison of the relative abundance of each metabolite in groups was further validated using the *p* values from two-tailed Student’s t-test. Each dot represented a metabolite, blue denoted downregulated ones, and pink represented those upregulated in smoking HTN. The value of VIP was expressed as the dot size. Among the varied metabolites between groups, those successfully identified were further labeled with names. **(C, D)**, Hierarchical cluster analysis heat-maps of identified metabolites with significant disparate levels between smoking HTN patients and non-smoking controls. The relative abundance of each metabolite in each individual is depicted.

To evaluate the detailed differences in metabolome between groups, relative abundance of each metabolite was analyzed. A Supplementary Table S1 reporting both nominal p-value and FDR has been provided (Supplementary Table S1). The detailed information for annotation levels of the annotated features has been described in Supplementary Table S2. As shown in Figures 2A,B, all the metabolic features detected were depicted with fold change of abundance between HTN-S and HTN-NS, VIP of OPLS-DA model in the multivariate statistical analysis, and p values in the univariate analysis. We observed 184 apparently increased and 421 reduced metabolicfeatures based on univariate analysis in HTN-S as compared with non-smoking patients, with 186 and 235 decreased under ES+ and ES- mode, 57 and 127 elevated under ES+ and ES- mode, respectively (Figures 2A,B).

Among these compounds, features with VIP scores >1 were considered as significant different in HTN-S, and those successfully identified metabolites were labeled in the volcano plots. These serum metabolites with large discrepancy between HTN-S patients and non-smoking controls were further visualized in heat-map (Figures 2C,D). Z-score comparison for these differentially abundant metabolites was performed in each individual (Supplementary Figures S1A, B). Of note, compared with non-smoking subjects, we found that most serum metabolites under ES- were depleted in HTN-S, such as 20-Hydroxyeicosatetraenoic acid, 1-Stearoyl lysophosphatidic acid, Hippuric acid, Glyceric acid and Glutaric acid, and most serum metabolites in ES+ were less abundant in hypertensive patients with tobacco consumption, e.g., N-Acetyl-L-aspartic acid, L-Pipecolic acid, Phenylacetic acid and Capric acid, etc.

**FIGURE 3:**
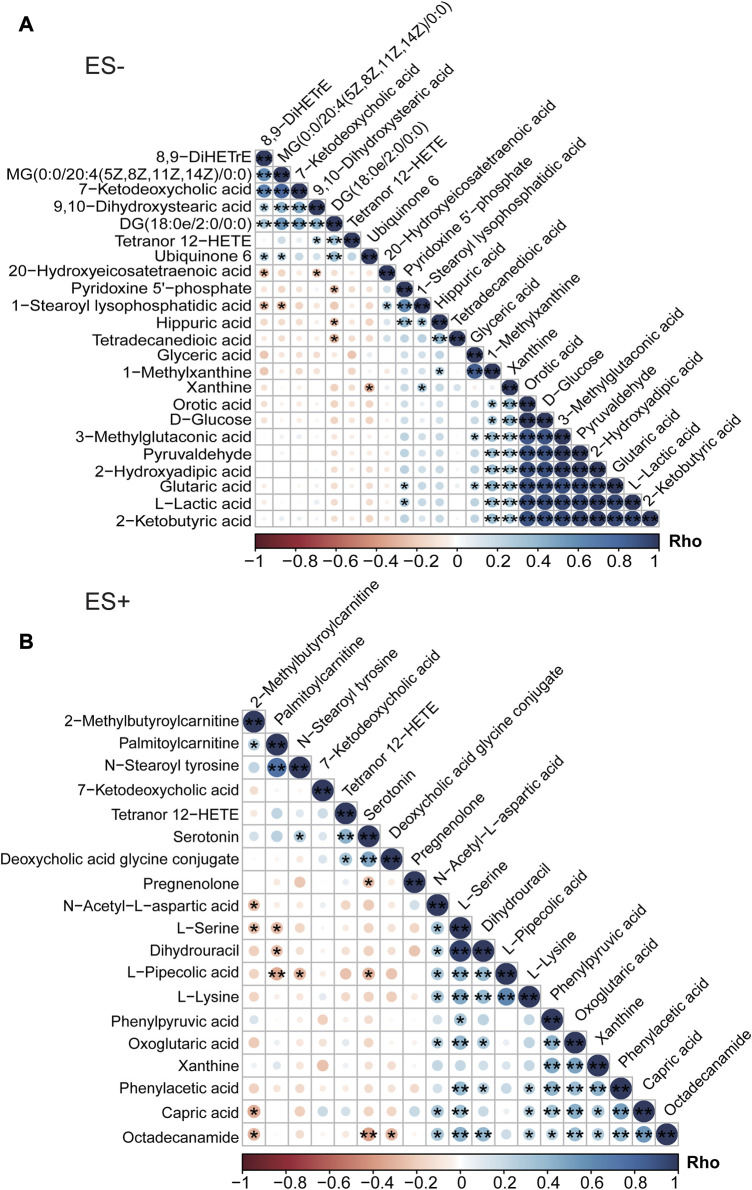
Association analysis of differential metabolites between HTN-S patients and HTN-NS controls by Spearman correlation. **(A)**, Co-abundance correlation of the distinct metabolites identified in HTN-S as compared with HTN-NS based on ES- mode. **(B)**, Heat map depicting the potential relationship between different metabolic compositions in ES+. Negative correlation is described in orange and positive correlation is labeled in blue. *, *p* < 0.05; **, *p* < 0.01; ***, *p* < 0.001; Spearman’s correlation.

Additionally, dramatic co-abundance correlations were revealed among these discriminative metabolites (Figures 3A,B). For instance, Glutaric acid was positively related with Pyridoxine 5′-phosphate, Glyceric acid, Orotic acid and 2-Hydroxyadipic acid. Especially, more profound association between metabolites was observed for those under negative mode. Thus altogether, these findings suggest that HTN patients with smoking behavior exhibit significantly different metabolic profiles compared with those of non-smoking subjects.

### 3.4 Pathway enrichment analysis of discrepant metabolites

**FIGURE 4:**
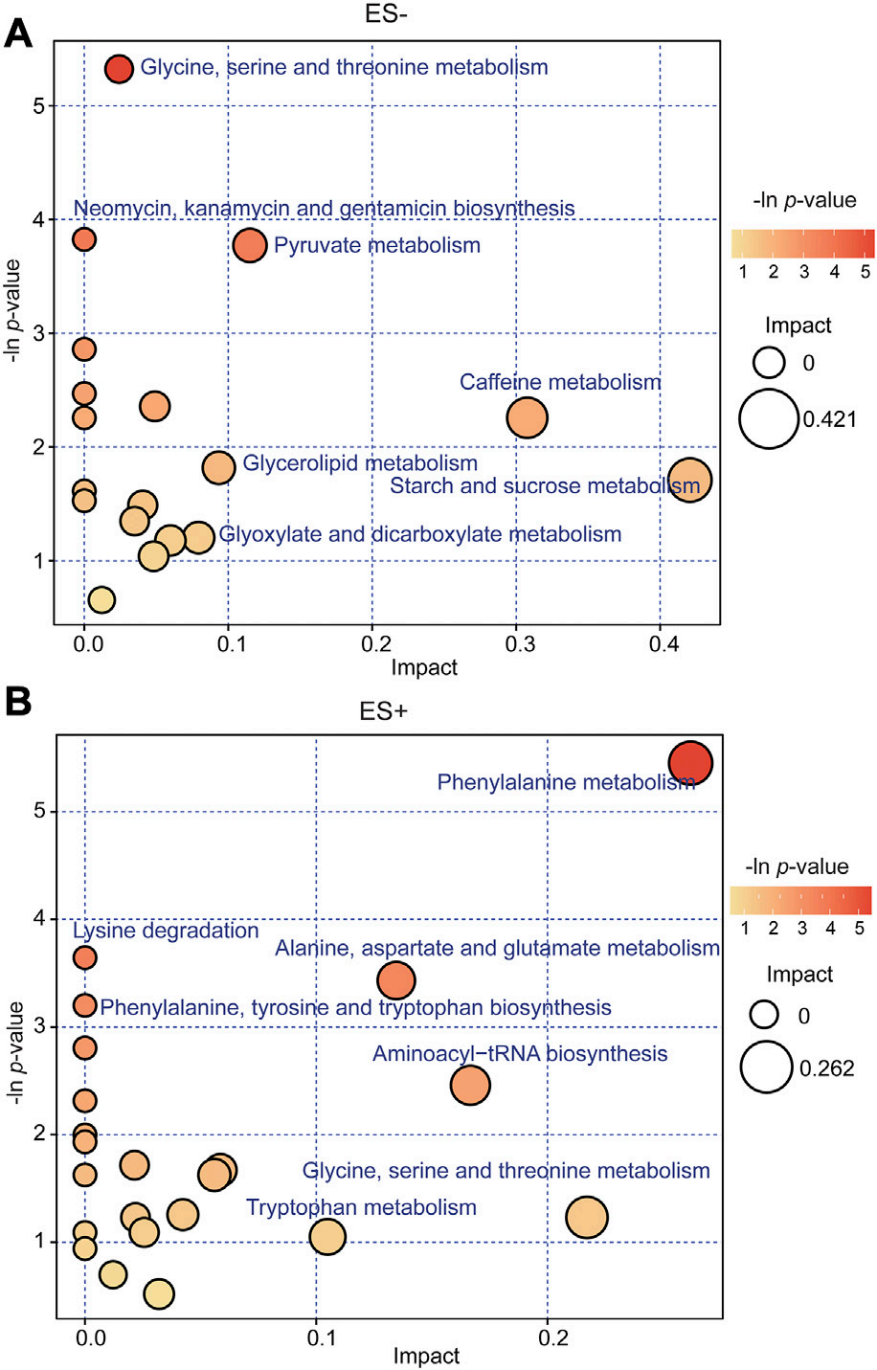
Kyoto Encyclopedia of Genes and Genomes (KEGG) pathway analysis of the differentially expressed compounds for group HTN-S vs. HTN-NS. **(A, B)**, Bubble plots in ES− and ES + showing the enriched metabolic pathways of varied metabolic compounds between groups, respectively. The color and *Y*-axis of dots are based on the -lnP-value, and the enrichment degree is more significant when the color is darker. The size and *X*-axis of dots represent the impact factor of the pathway in the analysis.

Concerning the main metabolic pathways and signaling pathways that the differential metabolites participated in, KEGG enrichment analysis was performed, and potential functions wer determined. In order to more comprehensively describe the functional capacities of metabolites and the pathways they participate in, we conducted analysis to explore the crucial pathways these metabolites involved in. The distinct metabolites between groups are labeled and visualized in KEGG pathway map, where enhanced metabolites were in red and depressed ones in blue (Supplementary Figure S2). Each metabolite was assigned to the corresponding KEGG pathway it acts in. The detailed information for pathways those discrepant metabolites matched to, including pathway name, the total number of all metabolites within each pathway, the number and name of differential metabolites matched in each pathway, *p* values and impact was shown in Supplementary Table S3. Within KEGG database, the distinct metabolites between groups were identified to be involved in multiple pathways regarding Glycine, serine and threonine metabolism, Neomycin, kanamycin and gentamicin biosynthesis, Pyruvate metabolism, Phenylalanine metabolism and Alanine, aspartate and glutamate metabolism, etc. (Figures 4A,B). There were 10 differential metabolites enriched in Phenylalanine metabolism, and 33 metabolites in Glycine, serine and threonine metabolism, which matched with more differential metabolites than the other pathways. Moreover, the pathways of Starch and sucrose metabolism and Phenylalanine metabolism exhibited higher impact in the analysis under ES- and ES+, respectively. Phenylalanine metabolism and Glycine, serine and threonine metabolism were the most significant metabolic pathways these metabolites functioned on.

### 3.5 Associations among serum metabolites and intestinal microbiota and clinical indicators

**FIGURE 5:**
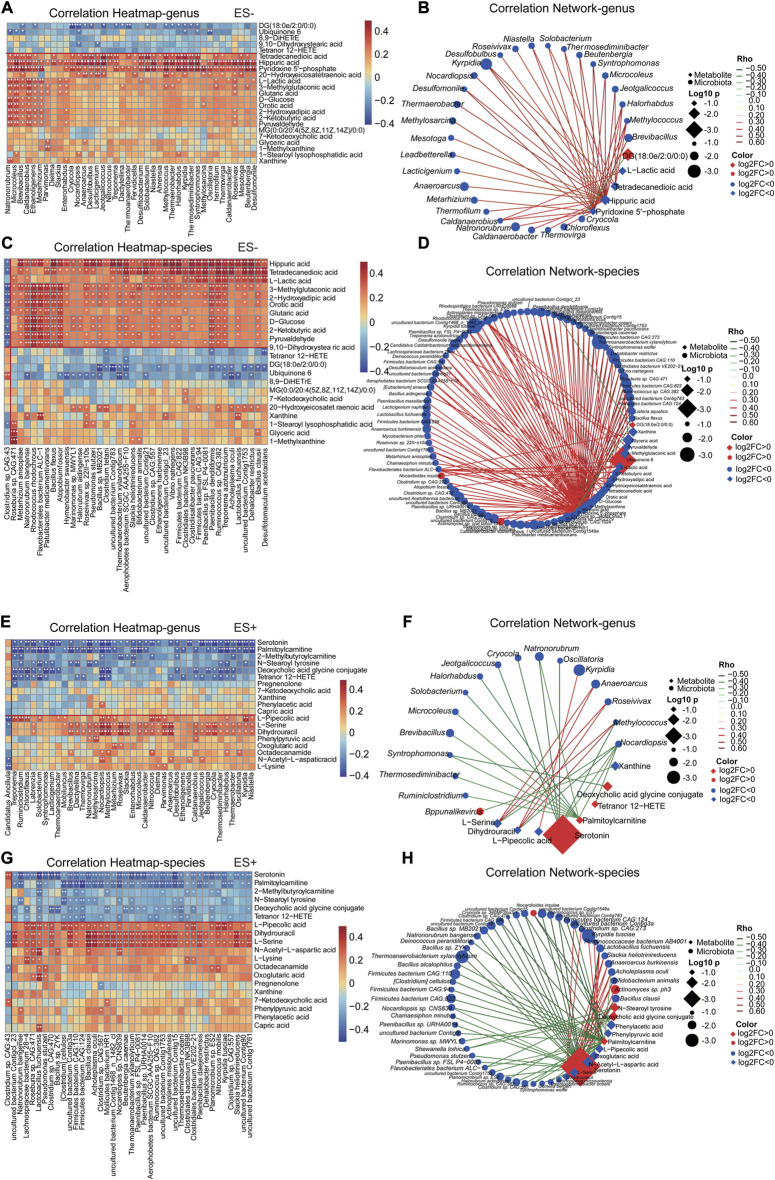
Smoking HTN-associated serum metabolites correlated with gut microbial genera and species differentiating HTN-S vs HTN-NS individuals. **(A, C, E, G)**, The association of gut microbial genera and species with top 40 serum metabolites in the study cohort was described with heatmap. The genera, species and metabolites included were those identified as significantly disparate between HTN-S vs. HTN-NS samples. A is for correlation of genera and metabolites, and C is association of species with metabolites, respectively. Positive associations are in red, and negative associations are in blue. *p < 0.05, **p < 0.01, and ***p < 0.001, Spearman’s rank correlation. **(B, D, F, H)**, Correlation network was produced based on integration of microbiome and metabolome datasets. Differential microbial variances were highly linked with differential metabolites. The correlation coeffcient is ≥ 0.4 or ≤ −0.4, and *p* < 0.05, calculated from Spearman correlation. Color of the nodes has been updated to represent log2FC, and different data type for metabolite/clinical/microbes is denoted with the node shape. Log10 *p*-values is described with node size to highlight the major correlations, and spearman Rho was displayed using continuous scaling of coloring of the edges. The datatype was used for attribute circular layout to group each category.

In order to assess the relationships and explore the potential interaction of altered serum metabolites, gut microbiota profiles and clinical parameters in participants, Spearman’s correlation analysis was conducted subsequently. Discriminative genera and species between hypertensive smokers and HTN-NS (Wang et al., 2021) were reanalyzed and evaluated in the present study. The association of top 40 differential with the top 40 distinct intestinal genera and species, respectively were shown in heat-maps (Figure 5).

These metabolites were detected to be strongly related to the abundance of gut microbiota. For instance, Pyridoxine 5′-phosphate and Hippuric acid exhibited a significantly positive correlation with *Natronorubrum*, *Microcoleus*, *Thermaerobacter*, *Halorhabdus* and *Oscillatoria*, etc. Serotonin and Palmitoylcarnitine were prominently negatively associated with *Ruminiclostridium*, *Chloroflexus*, and *Oscillatoria*, etc. It was interesting that, Pyridoxine 5′-phosphate, Hippuric acid and T etradecanedioic acid showed a significant correlation with most discriminative genera and species. Co-abundance network in Figures 5B,D,F,H further showed that Pyridoxine 5′-phosphate and Hippuric acid were positively linked to a large cluster of fecal bacteria, such as *Thermococcus* sp. *ES1*, *Clostridium* sp. *CAG:557*, *Bacillus clausii*, *Caldatribacterium saccharofermentans*, *Lactobacillus fuchuensi* and so on, indicating Pyridoxine 5′-phosphate and Hippuric acid might be potential gut microbiome relevant small-molecule products.

**FIGURE 6:**
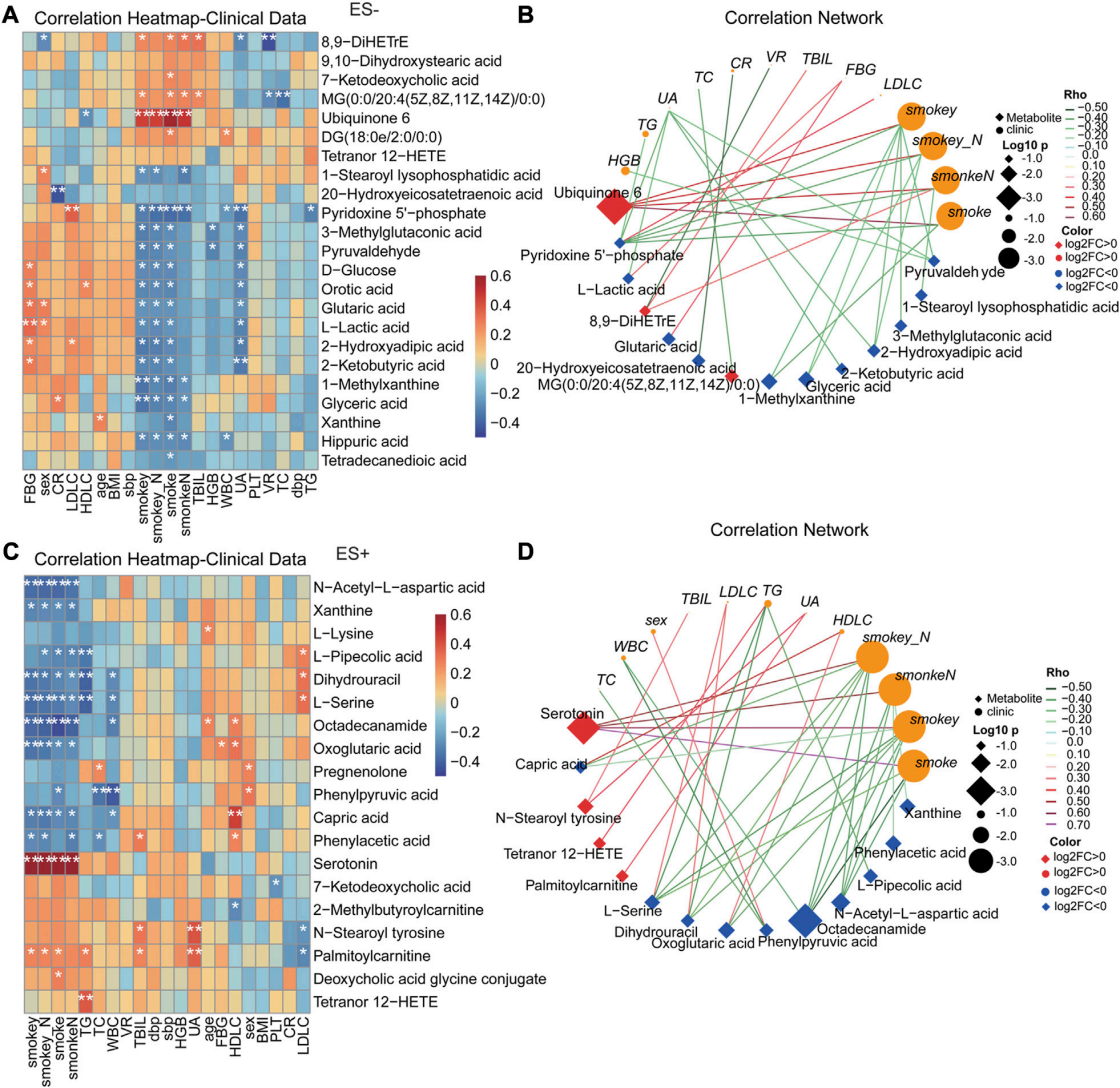
Correlation between clinical indexes of subjects and the important serum metabolites altered specifically in smoking HTNs. **(A, C)**, Heat map of the Spearman’s rank correlation coefficient of differential serum metabolic compounds and clinical indexes. Red squares indicate positive associations between metabolites and clinical indexes; blue squares indicate negative associations. The statistical significance was labeled with *p < 0.05, **p < 0.01, respectively. **(B, D)**, Correlation network describing the significant linkage between metabolites and clinical parameters. The correlation coefficient is ≥ 0.3 or ≤ −0.3, and *p* < 0.05, tested by Spearman correlation. Color of the nodes has been updated to represent log2FC, and different data type for metabolite/clinical/microbes is denoted with the node shape. Log10 *p*-values is described with node size to highlight the major correlations, and spearman Rho was displayed using continuous scaling of coloring of the edges. The datatype was used for attribute circular layout to group each category. SmokeN: smoking amount (cigarette/day); smokey: smoking duration (year); smokey*N: smoking coefficient (year cigarette/day).

Analogously, as shown in Figure 6, most of clinical features were significantly correlated with altered serum metabolome in HTN-S. Particularly, smoking index were dramatically positively associated with Ubiquinone 6, 8,9−DiHETrE and Serotonin, but negatively related with Glyceric acid, Orotic acid, Pyridoxine 5′-phosphate, Capric acid, Octadecanamide and N-Acetyl-L-aspartic acid (Figures 6A,C). Co-variation between the altered serum metabolites and clinical parameters exhibited significant and complicated association (Figures 6B,D). LDLC was positively linked to Pyridoxine 5'−phosphate, Dihydrouracil and L−Serine, Fasting blood glucose with L−Lactic acid and Glutaric acid, and conversely smoke showed negative association with Pyridoxine 5′-phosphate, 1-Methylxanthine and Octadecanamide, implying a possible contribution of metabolites to the host.

### 3.6 Random forest classifier identifying HTN-S with metabolic and microbial biomarkers

**FIGURE 7:**
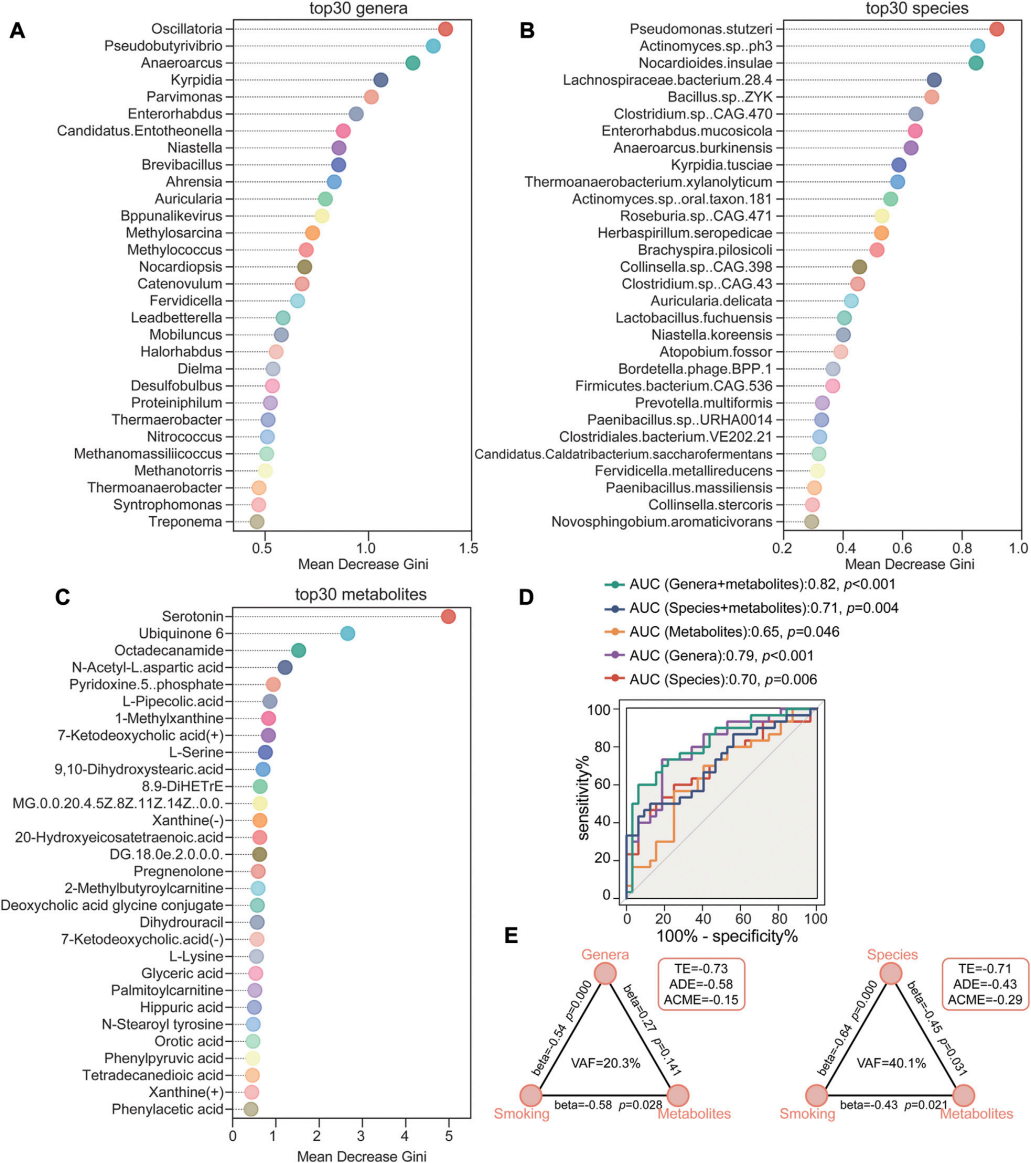
Random forest classification based on gut microbiome and metabolites to identify HTN-S patients from HTN-NS controls. **(A–C)**, Random forest models were conducted respectively to evaluate the potential of fecal genera/species and serum metabolites to discriminate between HTN-S and HTN-NS. The top 30 most distinguishing genera/species and metabolites between HTN-S and HTN-NS in the random forest analysis. Mean decrease Gini shown on the *x*-axis indicates the importance of each variable (genera, species or metabolites) for the classification. **(D)**, Receiver operating characteristic (ROC) curves showed the sensitivity, specificity and area under the curve (AUC) with explanatory variables of the top 30 most distinguishing genera, species or metabolites to distinguish smoking HTN patients from non-smoking individuals. **(E)**, The mediation effects of top 30 most distinguishing gut genera/species (indirect effect) on the total effect of smoking on top 30 metabolites. Path coefficients (beta) are labeled beside each path and indirect effect (VAF score) are denoted below each mediator variables. TE, total effect; ADE, average direct effect; ACME, average causal mediation effect.

To further explore the potential biomarker signatures of microbiome and metabolome for distinguishing smoking hypertensive patients, we conducted random forest disease classifier using the relative genera, species and metabolites abundances as variables, respectively. On the basis of the feature importance for the random forest model, as measured with the mean decrease Gini, we obtained ranked lists of metabolic and microbial features crucial for HTN-S. The top 30 most discriminatory microbial biomarkers were primarily from genus Oscillatoria, Pseudobutyrivibrio, Anaeroarcus, Kyrpidia, Parvimonas, and species *Pseudomonas stutzeri*, *Actinomyces sp. ph3*, *Nocardioides insulae*, Lachnospiraceae *bacterium.28.4*, *Clostridium.* sp. etc. (Figures 7A,B). And serum metabolites such as Serotonin, Ubiquinone 6, Octadecanamide, N-Acetyl-L-aspartic acid, Pyridoxine-5-phosphate and L-Pipecolic acid contributed the most to discriminate HTN-S from HTN-NS (Figure 7C). We implemented receiver operating characteristic (ROC) curves to evaluate the discriminative values of variables including top30 gut genera, species and serum metabolites. It showed an area under the curve (AUC) of species = 0.70 (*p* = 0.006), genera = 0.79 (*p* < 0.001), metabolites = 0.65 (*p* = 0.046), species + metabolites = 0.71 (*p* = 0.004), and genera + metabolites revealed an AUC of 0.82 (*p* < 0.001) (Figure 7D). Comparing to the other variables, the variable of genera + metabolites was more effective to classify HTN-S samples from HTN-NS. For metabolite and microbial biomarkers identified in the present study, we have performed a validation in the form of out-of-bag error for the random forest predictive model. Out-of-bag error rate of the random forest model with variables of the top 30 most distinct genera, species and metabolites to distinguish smoking HTN patients from non-smoking individuals is 0.258, 0.194 and 0.113, respectively (Supplementary Figures S3A–C).

Based on the crosstalk among smoking, gut microbiome and metabolites in HTN, we employed PLS-SEM to test the mediation effects of gut genera and species (indirect effect) on the total effect of smoking on metabolites (Figure 7E). The VAF score was used to estimate the proportion of indirect effect to total effects, and a VAF score at 20%–80% suggests a partial indirect effect. The mediation model evaluating the strength of the indirect effects, identified that the direct relationship between smoking and metabolites was statistically significant, and for indirect effect, the effect of species was statistically significant. The VAF for genera and species between smoking and metabolites was 20.3% while that of the species was 40.1% (Figure 7E). Thus, the contribution of smoking to serum metabolome was partially mediated by influencing the gut microbiome composition.

## 4 Discussion

In the current study, we identified profound association between cigarette smoking status and serum metabolomic profiles among hypertensive patients. Both PLS-DA and OPLS-DA models derived from untargeted metabolomics analysis showed significant discriminations in metabolic profiles and characteristics between smoking HTN patients and the non-smoking controls. Interestingly, it was noted that the majority of discrepant serum metabolites obtained, such as L-serine, Pipecolic acid, L-Lactic acid, were significantly deficient in cigarette smokers subjected to HTN. Furthermore, a classifier based on intestinal microbiota at genus level and core metabolites was established to accurately distinguish smokers from non-smokers among hypertensive patients.

It has been widely recognized that smoking and HTN are both crucial risk factors for cardiovascular diseases (Hopkins and Williams, 1986). Previous studies demonstrated that the combination of HTN and current smoking would extert an additive effect on the risk of developing cardiovascular and cerebrovascular diseases (Huangfu et al., 2017; Hara et al., 2019). Specifically, Huangfu et al. have shown that the cumulative incidence rate of ischemic stroke was 0.85%, 2.05%, 3.19% and 8.14% among non-HTN/non-smokers, non-HTN/smokers, HTN/non-smokers, and HTN/smokers, respectively. In addition, participants with coexistence of cigarette smoking and HTN were suggested to be at the highest risk for ischemic stroke disorders (Huangfu et al., 2017). Investigators reported that HTN and current smoking had a synergistic effect on the risk of progressing from moderate to severe cerebral small vessel diseases with OR: 10.59, 95% CI: 3.97–28.3, and synergy index: 4.03, 95% CI: 1.17–28.3) (Hara et al., 2019).

The gut microbiome is known to exert a vital impact on host physiology, and emerging evidence have successively confirmed that gut microbiota dysregulation is closely implicated in the occurrence and development of cardiovascular diseases and metabolic disorders (Tilg and Kaser, 2011; Tang et al., 2017). Previously, researchers have described the intestinal microbial dysibosis in smoking hypertensive patients, which is mainly manifested with decreased α-diversity and inclined to *Provotella*-dominant type (Wang et al., 2021). Simultaneously, in cigarette smokers suffering HTN, dramatic alterations of intestinal genus and species composition were detected, such as reduced enrichment of *physphaera* and *Clostridium asparagiformme*, etc. The fecal microbial metabolites are believed to induce host responses in the intestine and even at the distant organs (Liu et al., 2022). The metabolite profiles facilitates us to explain a high proportion of the varied functions for gut microbiome and thus have been regarded as intermediates that mediate the host-microbiota crosstalk (Zierer et al., 2018; Visconti et al., 2019). To the best of our knowledge, here we for the first time examined the serum metabolome of smokers with HTN, and further explored the correlation with gut microbiome.

L-pipecolic acid is known as a cyclic amino acid derived from L-lysine (Pérez-García et al., 2019). Previous studies indicated that the excessive accumulation of pipecolic acid is associated with host disorders of Zellweger syndrome, chronic liver diseases and pyridoxine-dependent epilepsy, etc. (Yu et al., 2020). Moreover, Yuan X and colleagues showed that, when compared to ulcerative colitis patients without depression/anxiety, those with ulcerative colitis and depression/anxiety subjects exhibited much lower abundance of L-pipecolic acid. It was most attractive that, prophylactic administration of L-pipecolic acid was identified to significantly reduce depressive-like behaviors in mice with colitis and prominently alleviate inflammatory cytokine levels in their colon, blood and brain (Yuan et al., 2021). Similarly, in the present work, we also observed apparent decrease in the abundance of L-pipecolic acid among smoking HTN patients. Further we revealed that L-pipecolic acid was positively interacted with butyrate-producing bacteria such as *Clostridim spp.* (*Clostridium* sp. *CAG:470*; *Clostridium* sp. *CAG:557*), *Ruminococcus spp.* (*Ruminococcus sp CAG:382*) and *Bacillus clausii*. Butyrate-producing bacteria are considered as a group of beneficial bacteria that produce butytric acids through fermenting dietary fiber (Nylund et al., 2015). For example, the Gram-positive gut bacteria *Ruminococcus*, has been demonstrated to be quite enriched under health status but markedly depleted in numerous diseases including human motor neuron disease (Rowin et al., 2017; Saad et al., 2021). Besides, *Bacillus clausii* was verified to possess immunomodulatory activity, and play important role in regulating cell growth and differentiation, cell adhesion, signal transcription and transduction, vitamin production and protection of the intestine from genotoxic agents. Therefore, in recent years, preparations containing *Bacillus clausii* have been frequently applied in the treatment or prevention of gut barrier impairment (Lopetuso et al., 2016).

L-serine is generally regarded as non-essential amino acid, but the term “conditionally non-essential amino acid” might be more appropriate for it, since under some circumstances, vertebrates are unable to produce it with sufficient quantities to meet the necessary cellular requirements (Metcalf et al., 2018). It was reported that compared with the mice that compromised from *Klebsiella pneumonia* lung infection, enrichment of L-serine was detected in mice that survived during the infection and L-serine was indicated to be associated with the host surviving. Furthermore, L-serine was able to facilitate macrophage phagocytosis, and participate in a natural way to promote host clearance of lung pathogens (Liu et al., 2018). In addition, it was shown that the level of L-serine was also significantly reduced in murine lungs infected with *Pasteurella multocida*. Exogenous supplementation of L-serine would significantly enhance the survival rate of infected mice and suppress the colonization of *Pasteurella multocida* in mice lungs (He et al., 2019). Our findings that the abundance of serum metabolite L-serine, was depleted in smokers with HTN, is in agreement with the changes previously observed in other lung diseases (Liu et al., 2018; He et al., 2019).

Actually, several pervious studies have reported the serum metabolomic profiles between essential hypertension and healthy controls (Dołegowska et al., 2009; Shi et al., 2022; Sun et al., 2022). Metabolites such as 2-methylbutyrylcarnitine (Shi et al., 2022) and 12-HETE (Dołegowska et al., 2009) have been demonstrated to be dramatically enriched, while L-Serine (Shi et al., 2022; Sun et al., 2022) was depleted in hypertensive patients in comparison with normotensive controls. It is worth noting that our findings in the current work confirm that the abundance of these serum metabolites discrepant between normal individuals and hypertensive patients, including enhanced 2-methylbutyrylcarnitine and Tetranor 12-HETE, and suppressed L-Serine were further more severely altered in hypertensive smokers.

Cotinine is the utmost important nicotine metabolite (Rolle-Kampczyk et al., 2016). Benowitz and his colleagues indicated that cotinine has been proved to be a suitable marker to differentiate smoke burdened from unburdened persons (Benowitz, 1996; Benowitz et al., 2009). Furthermore, Rolle-Kampczyk UE et al. found that urine cotinine levels were significantly higher among mothers who smoked during pregnancy in comparison with non-smokers (Rolle-Kampczyk et al., 2016). The metabolite cotinine was not detected in the present study, which might be due to the different study population, sample collection site and metabolome detection methods. In addition, Gu F et al. reported that both cotinine and serotonin were positively correlated with current smoking status in a cohort from the Environment And Genetics in Lung Cancer Etiology study (Gu et al., 2016). Although metabolite cotinine was not detected to be discrepant in the present study, we identified higher level of serotonin in smoking HTN patients, which confirmed previous study to a certain extent (Gu et al., 2016).

Previous studies have elucidated the potential capacity of gut microbiome combined with metabolites as biomarkers for distinguishing various diseases, such as thyroid carcinoma, spontaneous preterm birth, Alzheimer’s disease, etc (Feng et al., 2019; Flaviani et al., 2021; Xi et al., 2021). Previously, a discriminate predictive model based on eight metabolites as well as five genera displayed excellent distinguishing effect between thyroid carcinoma patients and healthy controls (Feng et al., 2019). In the present study, we constructed a random forest classifier with the combination of 30 gut bacterial genera and 30 metabolites, which was able to discriminate HTN-S from HTN-NS with an AUC of 0.82. These results illustrated that biomarker signatures according to the gut microbiome and metabolome exert strong reliability in identifying smokers with HTN from non-smokers, which emphasized the significance of gut microbiota and metabolome. On the basis of the previous results, we also conducted mediation analysis, showing that the contribution of smoking status to the serum metabolome was partially mediated by affecting the composition of the intestinal microbiome.

Nevertheless, several limitations have to be acknowledged in the present study. Firstly, the number of participants was relatively small which might restrict the generalization of our results. Since population used to identify the biomarkers did not show any clinical, anthropometrical, or biochemical difference, further studies are still needed to validate these biomarkers in other hypertensive population with cardiovascular diseases, and a direct causal relationship among cigarette smoking, fecal microbiota, serum metabolites during HTN development is warranted to be elucidated in a cell/animal model thoroughly. Secondly, untargeted LC-MS was conducted when analyzing metabolite compositions in smoking and non-smoking HTN patients. Target mass spectrometry, which is more sensitive and more quantitative would also be necessary to further confirm the present findings. Lastly, we admit that the information regarding mode of birth and alcoholic drinking is lacking for the participants.

## 5 Conclusion

In summary, the findings based on this study demonstrated significant discrepancy in circulating blood metabolome profiles in HTN-S when compared with HTN-NS. A combination of gut bacterial genera and serum metabolites was capable to discriminate HTN-S from HTN-NS with good performance. In addition, the contribution of smoking to host metabolome was identified to be mediated at least partially by affecting the gut microbiome. Taken together, it is proposed that smoking cessation is extremely essential for hypertensive patients, which might be helpful to improve metabolic homeostasis and avoid future cardiovascular events by regulating gut microbiome and metabolites.

## Data Availability

The original contribution presented in the study are included in the article/Supplementary Material, the data presented in the study are deposited in the metabolights repository, accession number MTBLS 7057.
