# Supplementary figures and images for: Untargeted metabolomics unravel serum metabolic alterations in smokers with hypertension
Source: Front Physiol. 2023 Mar 2;14:1127294. doi: 10.3389/fphys.2023.1127294 (PMC10018148; doi:10.3389/fphys.2023.1127294)

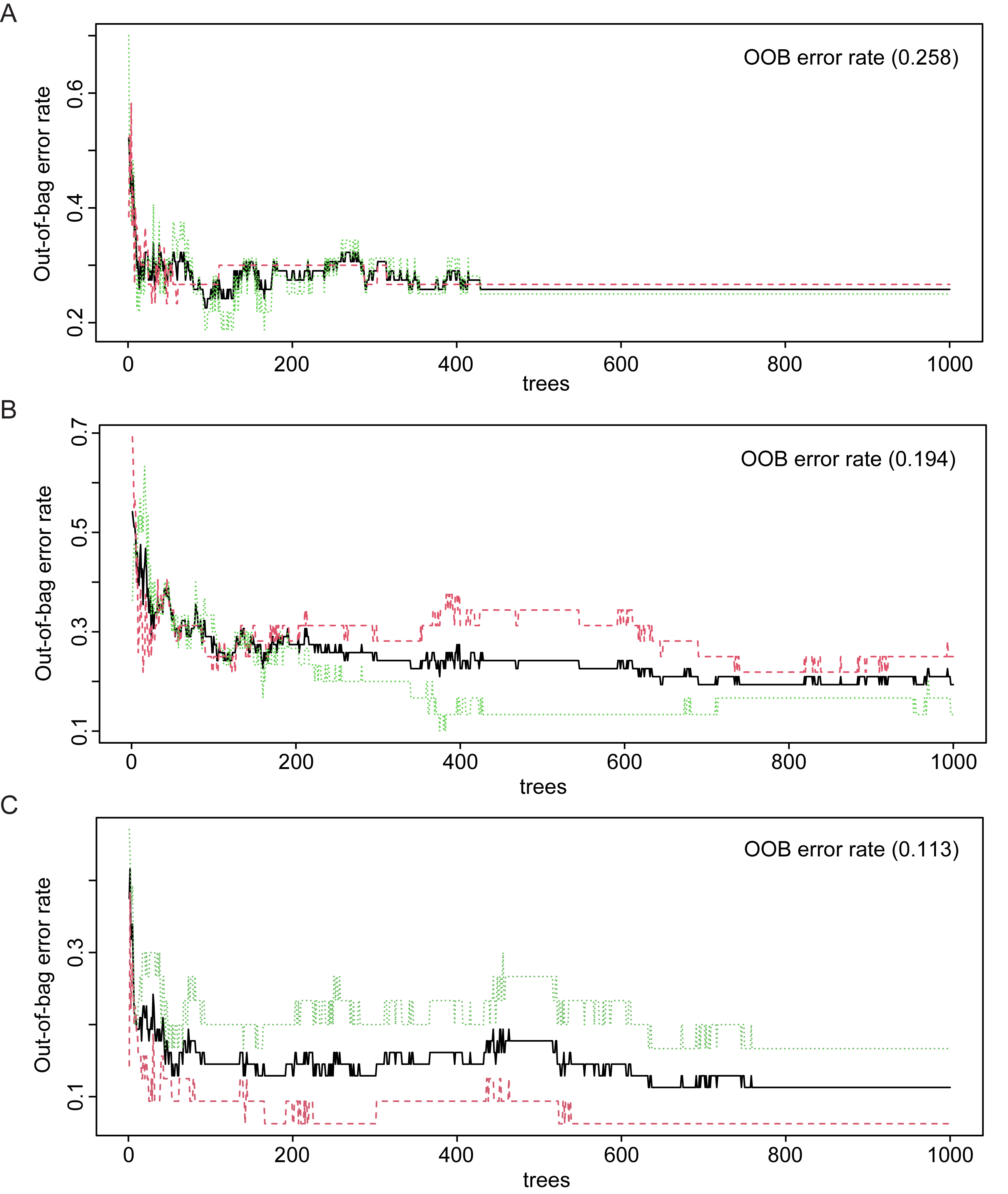

Supplement: Supplementary file 3 [file Image3.TIF]

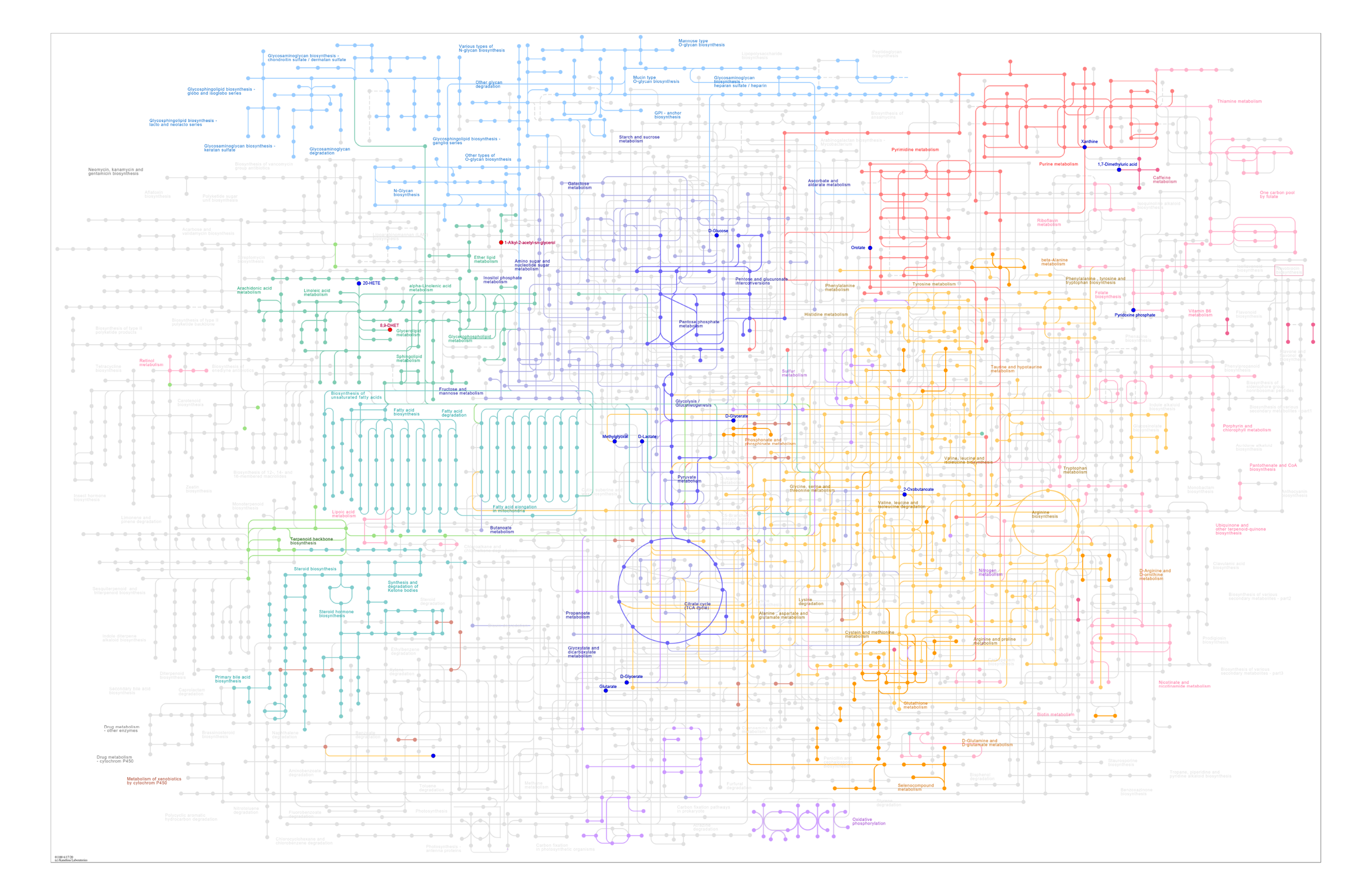

Supplement: Supplementary file 4 [file Image2.TIF]

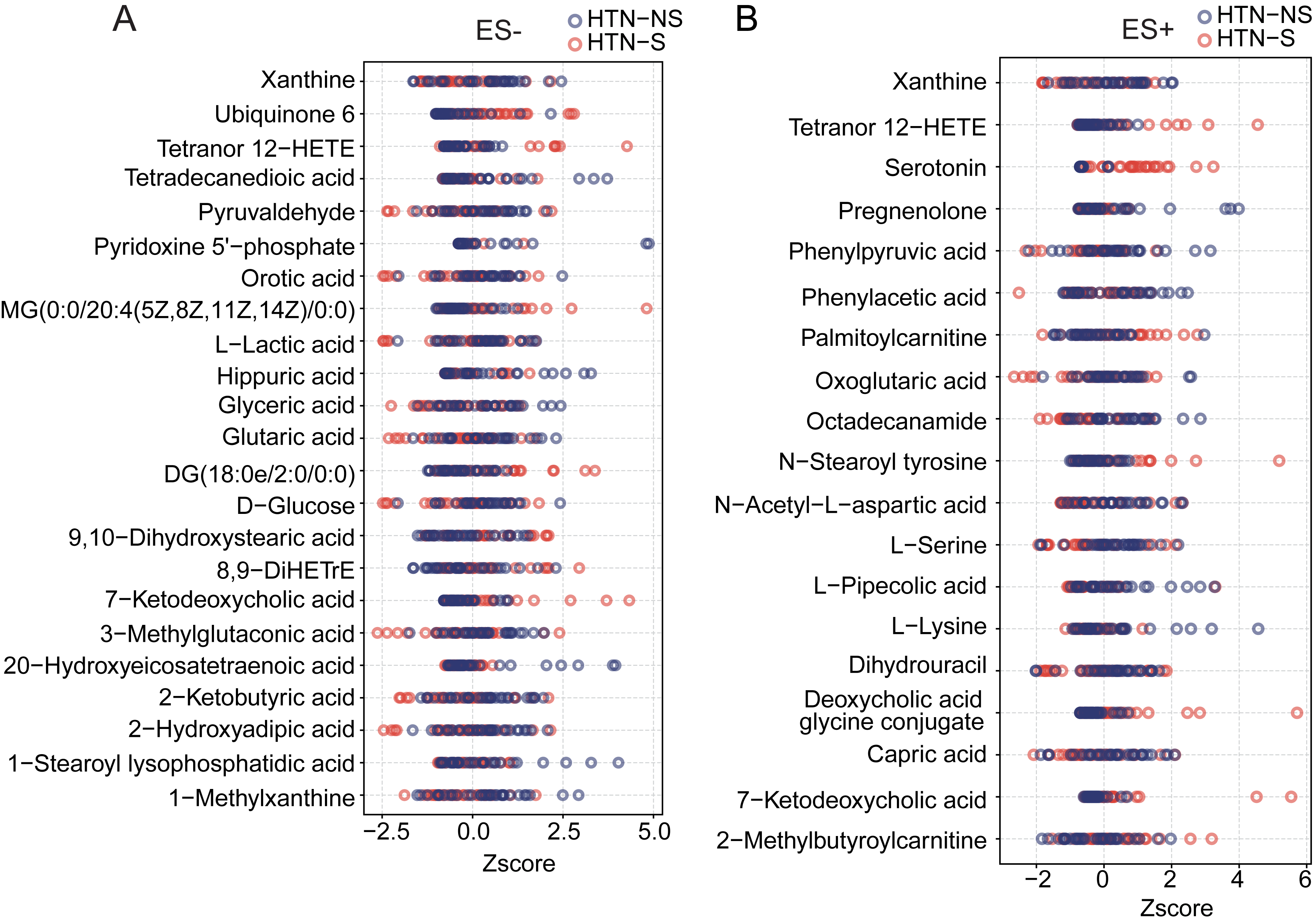

Supplement: Supplementary file 5 [file Image1.TIF]
